# Dynamic cutaneous information is sufficient for precise curvature discrimination

**DOI:** 10.1038/srep25473

**Published:** 2016-05-03

**Authors:** Jacob R. Cheeseman, J. Farley Norman, Astrid M. L. Kappers

**Affiliations:** 1 Department of Psychological Sciences, Ogden College of Science and Engineering, Western Kentucky University, Bowling Green, Kentucky, 42101-1030, USA; 2 MOVE Research Institute, Department of Human Movement Sciences, Vrije Universiteit, Amsterdam, Netherlands

## Abstract

Our tactual perceptual experiences occur when we interact, actively and passively, with environmental objects and surfaces. Previous research has demonstrated that active manual exploration often enhances the tactual perception of object shape. Nevertheless, the factors that contribute to this enhancement are not well understood. The present study evaluated the ability of 28 younger (mean age was 23.1 years) and older adults (mean age was 71.4 years) to discriminate curved surfaces by actively feeling objects with a single index finger and by passively feeling objects that moved relative to a restrained finger. While dynamic cutaneous stimulation was therefore present in both conditions, active exploratory movements only occurred in one. The results indicated that there was a significant and large effect of age, such that the older participants’ thresholds were 43.8 percent higher than those of the younger participants. Despite the overall adverse effect of age, the pattern of results across the active and passive touch conditions was identical. For both age groups, the curvature discrimination thresholds obtained for passive touch were significantly lower than those that occurred during active touch. Curvature discrimination performance was therefore best in the current study when dynamic cutaneous stimulation occurred in the absence of active movement.

In past research with objects that vary in 2-D shape^1^,^2^,^3^, active touch frequently produced higher identification or shape matching performance than static touch. For example, in an experiment by James Gibson^1^, a variety of “cookie cutter” objects (approximately one inch, or 2.5 cm, in diameter) were either pressed into a participant’s palm or were actively felt by participants using their fingers. When active exploration of the objects was allowed, the participants’ identification performance was 95 percent correct. In contrast, performance in the static conditions was either 49 or 29 percent correct, depending upon whether the objects were manually pressed into the participants’ palms by the experimenter (49% correct) or were mechanically applied using a lever (29% correct). In Experiment 3 of Norman *et al.*^4^, solid shape discrimination performance obtained with active haptic manipulation was compared to that obtained when the participants’ manual exploration was restricted. For naturally-shaped bell peppers, our participants’ performance was 16.6 percent higher in the active condition, whereas it was 14.6 percent higher for the “feelie” sculptures commissioned by Gibson (see his Fig. 7)^5^. Curvature discrimination is often better (e.g., lower thresholds) for active touch as compared to static or passive touch^6^,^7^,^8^. At the moment, it is not entirely clear exactly what information contributes to the superiority of performance that is frequently obtained during active haptic exploration. Some possibilities would include 1) dynamic cutaneous input (from mechanoreceptors in the skin), 2) kinesthetic input (from muscle, tendon, and/or joint receptors), and 3) corollary discharge (i.e., efference copy) within the central nervous system/brain that occurs in conjunction with exploratory movements.

On average, older adults possess lower tactile acuity than younger adults^7^,^9^,^10^,^11^. For example, using a tactile grating orientation discrimination task, Norman *et al.*^7^ found that the thresholds of older adults were two to three times higher than those of younger adults. In addition, older adults have been found to perform more poorly for static curvature discrimination^7^. It is therefore interesting to note that older adults frequently perform as well as younger adults for curvature or shape-related tasks where dynamic touch, or active haptic exploration, is allowed^7^,^9^,^12^,^13^,^14^. What is responsible for older participants’ good performance during active touch conditions? Is it the presence of dynamic cutaneous input *per se* (e.g., stimulation of the cutaneous mechanoreceptors of the fingers and hand)? Does the good performance depend upon kinesthesis and/or efference copy and thus require actual hand/arm movement? The purpose of the current experiment was to answer such questions.

## Method

### Apparatus

A custom-built apparatus consisting of an electric motor and slider-crank mechanism was used to control the movement of the stimulus objects in one of the two experimental conditions. An Apple MacBook computer was used to randomly order the presentation of the experimental stimuli and record the participants’ responses.

### Experimental Stimuli

**Figure 1:**
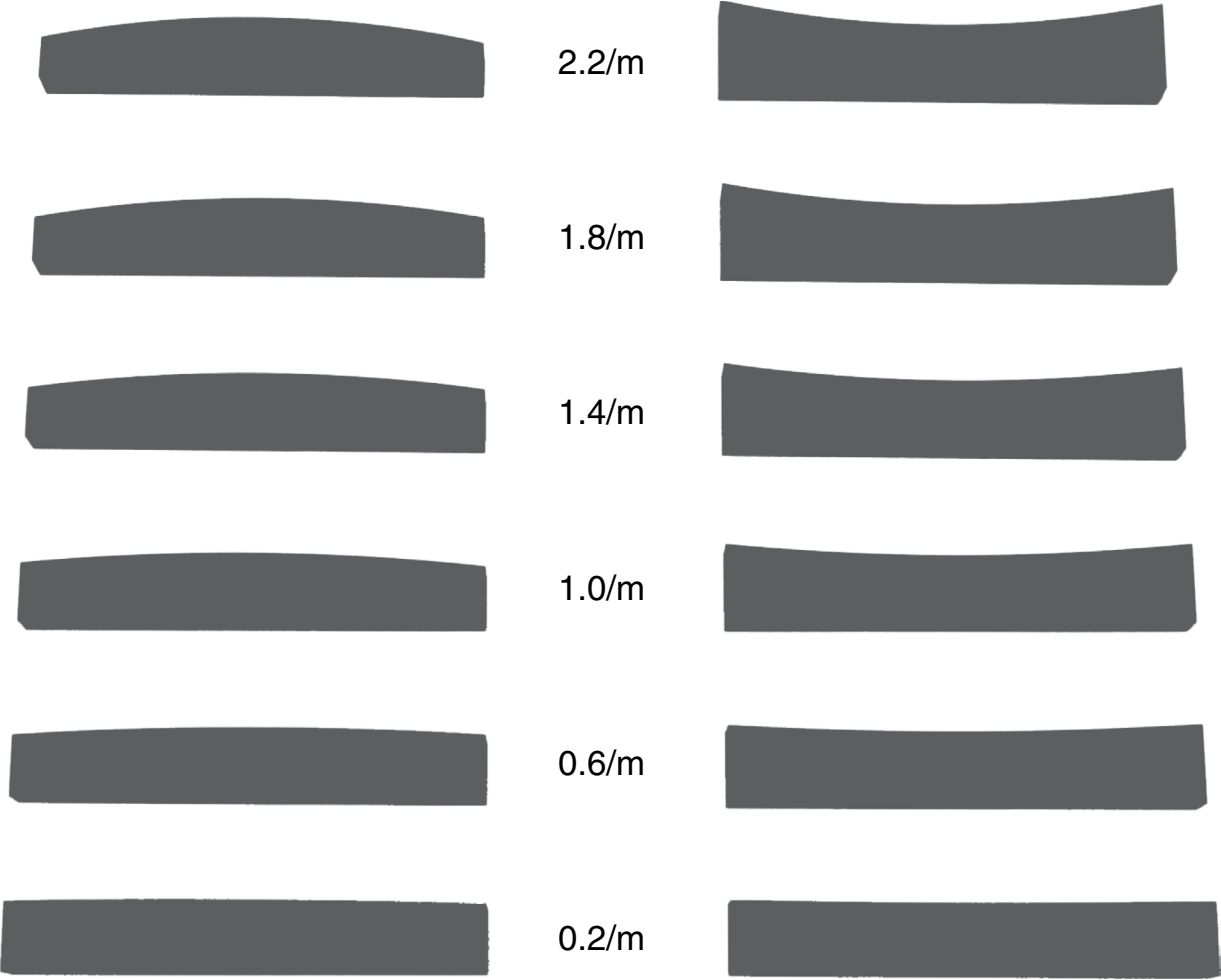
A side view of the curved blocks used as stimuli in the current experiment. The convex and concave stimuli are located at the left and right, respectively. The stimulus curvature magnitude decreases from the top of the figure downwards (2.2 to 0.2/m).

The stimulus objects were machine-milled polyvinyl chloride (PVC) plastic blocks (20 cm × 2 cm × ~5 cm) that have been used extensively in previous research^7^,^15^,^16^,^17^. The blocks featured convex or concave top surface curvatures, which ranged in magnitude from 0.2 to 2.2/m in increments of 0.4/m (see Fig. 1). Tactile gratings (JVP Domes, Stoelting, Inc.) with groove widths ranging from 0.75 to 6.0 mm were used to assess the participants’ tactile acuity. The 12 small objects used in the Moberg pick-up test of manual dexterity were the same as those used previously^9^,^12^.

### Procedure

**Figure 2:**
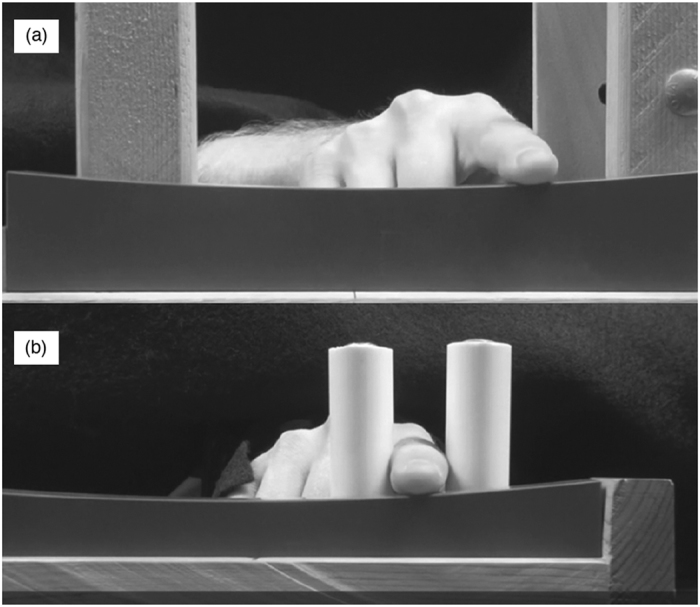
Photographs illustrating (**a**) active haptic curvature discrimination and (**b**) passive curvature discrimination. One can readily see that the participant’s haptic exploration of the stimulus block (concave) by the index finger is unhindered at top, while it is restricted from lateral movement at bottom. In the passive touch condition (**b**), the curved stimulus blocks translate underneath the stationary finger.

The basic procedures for the curvature discrimination task were similar to those used by Norman *et al.*^7^. On every trial, participants reached underneath an opaque curtain to feel the top surface of a stimulus block; they then judged its curvature to be either convex or concave. The participants performed this task using both active and passive touch. Each participant performed the touch conditions in an order that was counterbalanced across all participants (i.e., half of the participants used active touch first, while the remaining half used passive touch first). In the active touch condition (Fig. 2a), the blocks remained in a fixed position while the participants used their index finger to laterally explore their top surfaces. An aperture was used to limit exploration to the middle 10 cm extent of the blocks. The passive touch condition employed a procedure similar to that used by van der Horst *et al.*^17^ and Smith *et al.*^18^. As in the active touch condition, only the index finger contacted the blocks, but in this condition the blocks moved (i.e., translated) underneath and perpendicular to the long axis of the finger (see Fig. 2b). In order to prevent active manipulation, the participants’ hand, wrist, and arm were restrained into a fixed position; the participants’ index finger could only move up and down (to maintain contact with the block). After the participant’s hand and arm were secured, the blocks then translated ±5 cm relative to the fingertip at an average rate of 10 cm per second. Under these circumstances, the index finger passively felt the same 10 cm extent of the block that could be actively felt in the active condition.

The testing in each condition began with a block of trials evaluating each participant’s ability to discriminate convex and concave curvature magnitudes of 2.2/m. Subsequent blocks of trials evaluated discrimination of curvature magnitudes in descending increments of 0.4/m (e.g., curvature magnitudes of 2.2, 1.8, 1.4, 1.0, 0.6, and 0.2/m). The order of presentation of concave and convex stimuli within each block was randomly determined, and there was an equal probability of presenting a convex or concave stimulus on any given trial. For each individual participant, discrimination performances above and below a d′^19^ value of 1.35 were obtained; these two d′ values were then used to calculate a threshold estimate (i.e., the curvature magnitude needed to discriminate at a d′ value of 1.35) using linear interpolation. In order to reduce the total number of trials to a manageable number, the participants initially completed blocks of 12 trials for each curvature magnitude. If a participant made 10 or more correct judgments, testing would begin again with a new block of trials devoted to the next smaller curvature magnitude. If fewer than 10 of the 12 trials with a given curvature magnitude were judged correctly, however, the participants would then complete a new block of 40 trials with the current curvature magnitude, and all subsequent curvature magnitudes would be tested with 40-trial blocks. This procedure was utilized for all curvature magnitudes except 0.2/m (i.e., the minimum curvature magnitude of the stimulus set), which was always tested with 40-trial blocks.

Given that aging is known to reduce participants’ tactile acuity^7^,^9^,^10^,^11^, it is certainly possible that this age-related reduction could influence performance for the current tactile curvature discrimination task. Tactile acuity was accordingly assessed for all participants using grating orientation discrimination^20^,^21^. Grating orientation discrimination is a widely used task that possesses significant advantages over traditional methods used to evaluate tactile acuity, such as the determination of two-point thresholds^7^,^9^,^22^,^23^. In our study, tactile gratings were applied to the distal pad of each participant’s index finger by the experimenter for one second; on each trial, the participants judged whether the orientation of the grooves of the grating was parallel or perpendicular to the long axis of the finger. The order of presentation (parallel vs. perpendicular) within a block was randomly determined, and there was an equal probability of either stimulus orientation on any given trial. For younger participants, the first block of 40 trials utilized a groove width of 3.0 mm. Subsequent blocks of 40 trials used gratings with smaller and smaller groove widths (e.g., 2, 1.5, 1.2, & 1.0 mm) until a participant’s discrimination performance dropped below a d′ value of 1.35. Threshold estimates for tactile grating orientation discrimination were then calculated in the same manner (linear interpolation) as described for surface curvature discrimination. The procedure for determining tactile acuity for the older participants was identical, except that the initial groove width was larger (e.g., 4–6 mm), since it is well known that older adults possess higher thresholds^7^,^9^,^24^.

In addition to tactile acuity, we evaluated the participants’ manual dexterity–any reduction in dexterity could potentially affect a participant’s ability to actively explore the stimulus blocks while performing the curvature discrimination task. To evaluate manual dexterity, we required the participants to complete a modified version of the Moberg pick-up test^25^,^26^. This task has been used previously to evaluate manual dexterity and hand function in both younger and older adults^9^,^12^,^27^. In this task, the participants picked up 12 small metal objects (e.g., nail, paperclip, coins, flat-head screw, etc.) and placed them inside a container as rapidly as possible, and the cumulative time required to place the objects in the container was recorded. People with no substantial deficits in manual dexterity can perform this task well without seeing the objects. The participants performed this picking-up task with and without vision. This test was performed twice, with the best performance (shortest overall time) being included in the analysis.

### Participants

Twenty-eight younger and older adults participated in the experiment. Fourteen of the participants were older (*M* = 71.4 years of age, *SD* = 4.9, range = 67 to 80 years) and fourteen were younger (*M* = 23.1 years of age, *SD* = 1.5, range = 21 to 25 years). All participants were naive regarding the purpose of the experiment. The study was approved by the Institutional Review Board of Western Kentucky University and was conducted in accordance with the Code of Ethics of the World Medical Association (Declaration of Helsinki). Each participant signed an informed consent document prior to testing.

## Results

**Figure 3:**
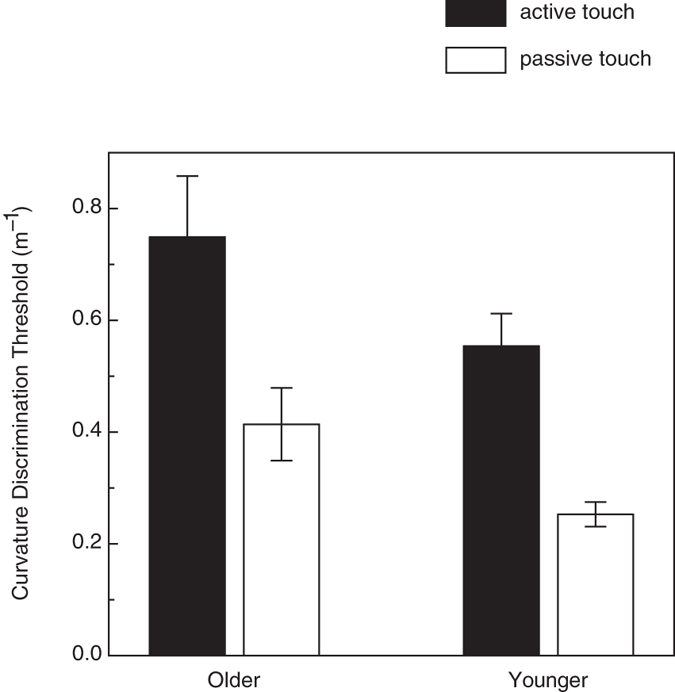
Experimental results. The younger and older participants’ curvature discrimination thresholds are plotted for both active and passive touch conditions. The error bars indicate ±1 SE.

Curvature discrimination thresholds for the two touch conditions for each age group are depicted in Fig. 3. Relative movement between the participants’ fingerpads and the stimulus objects occurred during both the active and passive touch conditions; therefore, dynamic cutaneous input was always present. In contrast, significant kinesthetic input/efference copy only occurred during active touch. According to a 2 × 2 factorial analysis of variance (ANOVA, age × touch type), the discrimination thresholds obtained for passive touch were significantly smaller than those obtained for active touch (F(1, 26) = 27.49, *p* < 0.001, η^2^_p_ = 0.51). When active touch was used, a higher curvature was required in order to reliably discriminate convex from concave surface shapes compared to when passive touch was used. In addition to the effect of touch condition, there was also a significant adverse effect of age (F(1, 26) = 5.0, *p* = 0.035, η^2^_p_ = 0.16): the older adults’ thresholds were 43.8 percent higher than those of the younger adults. There was no interaction between the effects of touch condition and age (F(1, 26) = 0.1, p = 0.78), thus the magnitude of the improvement exhibited by the older participants during the passive touch condition was just as large as that exhibited by the younger participants.

**Figure 4:**
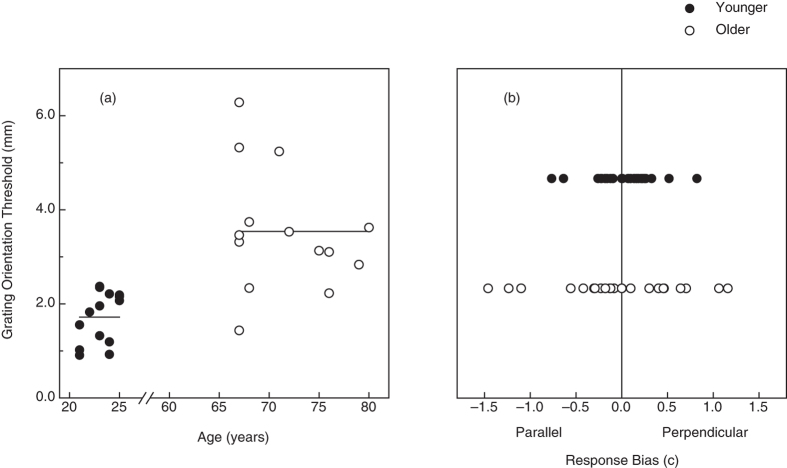
(**a**) Experimental results (Tactile acuity). The participants’ grating orientation thresholds are plotted for the younger (filled circles) and older (open circles) adults. The horizontal line segments indicate the mean threshold for each age group. (**b**) Experimental results (Response bias). The response biases (c values) exhibited during the grating orientation discrimination task (for blocks utilizing groove widths immediately above and below each participant’s threshold) are plotted separately for the younger (filled circles) and older (open circles) participants.

**Figure 5:**
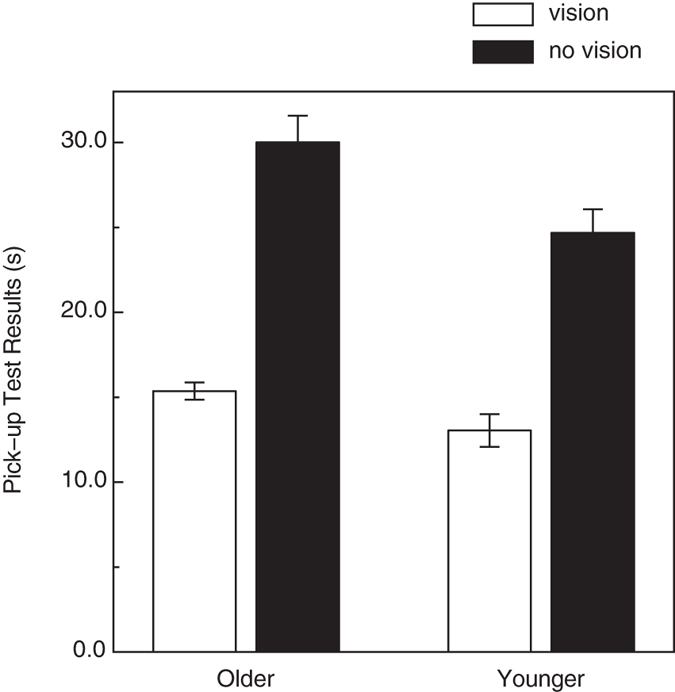
Experimental results (Manual dexterity). The results of the Moberg Pick-up Test are plotted for the younger and older participants. The cumulative pick-up times for the with- and without-vision conditions are indicated by the white and black bars, respectively. The error bars indicate ±1 SE.

Our older participants did have reduced tactile acuity compared to the younger participants (see Fig. 4a); their grating orientation discrimination thresholds (on average, 3.54 mm) were significantly higher (t(26) = 4.8, p < 0.0001, 2-tailed) than those obtained for the younger participants (1.72 mm). It is interesting that while there were significant effects of age upon both curvature discrimination and tactile acuity, there was no significant correlation between the participants’ tactile acuities and their performance for either active (Pearson r = 0.21, p = 0.29) or passive curvature discrimination (r = 0.29, p = 0.14). The participants’ response biases, c values^19^, that occurred during the grating orientation discrimination task are shown in Fig. 4b. One can readily see that when the older adults did have significant response biases (i.e., biases to respond either “parallel” or “perpendicular”), their response bias magnitudes were often larger than those exhibited by the younger adults (i.e., their absolute values of c were larger, F(1, 26) = 4.6, p < 0.05, η^2^_p_ = 0.15). In addition to assessing the participants’ tactile acuity, we also evaluated their manual dexterity; the results of the Moberg pick-up test are plotted for the younger and older participants in Fig. 5. According to a 2 × 2 ANOVA (age × vision condition), both main effects were significant (age: F(1, 26) = 6.9, *p* < 0.02, η^2^_p_ = 0.21; vision condition: F(1, 26) = 268.2, *p* < 0.000001, η^2^_p_ = 0.91), but not the interaction (F(1, 26) = 3.5, *p* > 0.07). The older adults’ average pickup times were 20.3 percent longer than those of the younger adults, reflecting their reduced manual dexterity. The participants’ curvature discrimination thresholds, however, did not correlate significantly with the dexterities obtained in either the with-vision condition (Pearson r’s were 0.04 and 0.26 for the active and passive touch conditions, respectively; p’s > 0.18) or the without-vision condition (Pearson r’s were .31 and 0.29 for the active and passive touch conditions, respectively; p’s > 0.1).

## Discussion

Our previous investigation^7^ found that dynamic curvature discrimination was superior to static curvature discrimination. When participants actively explore a stimulus object, they obviously obtain some type of information, whether cutaneous or kinesthetic/efference copy, that facilitates their perception of the object’s shape. The performance obtained in the current study during the active touch condition is consistent with this idea (the current active touch curvature discrimination thresholds were 32.6 and 17.1 percent lower than the static touch thresholds obtained by Norman *et al.*^7^ for older and younger adults, respectively). The current results additionally demonstrate, however, that tactual curvature discrimination performance is even better (even lower thresholds) during passive touch (see Fig. 3), where dynamic cutaneous stimulation is preserved but active finger movement is eliminated. It is therefore necessarily the case that it is the dynamic cutaneous information *per se* that is responsible for the good curvature discrimination performance that typically occurs in conjunction with active movement, and that kinesthetic information/efference copy is not responsible.

The current results, obtained for curvature discrimination of actual surface shapes, are analogous to those obtained by Smith *et al.*^18^ for virtual surfaces, where the convex and concave surface shapes were simulated by a device that manipulated (over time) the vertical displacement of participants’ fingers. Like us, Smith *et al.* found a superiority for passive over active touch (compare their Fig. 4a,b). In the study by Smith *et al.*, the participants’ finger always rested on a moveable plate and did not therefore touch a physically-curved surface; in actuality, their experimental setup simulated a participant touching a curved surface with a tool. Our results are similar to those of Smith *et al.* and show that their finding of a superiority for passive touch generalizes to real-world surfaces that are explored directly with the fingers.

The current results and those of Smith *et al.* demonstrate that passive touch can be superior to active touch in certain situations. How does this superiority occur; what is the potential mechanism? One likely possibility has been documented in neurophysiological experiments^28^,^29^,^30^,^31^. Chapman *et al.*^28^ found that neuronal responses to tactile stimuli (in monkey somatosensory cortex and the VPL nucleus of the thalamus) were suppressed during active arm movement. Seki *et al.*^29^ found that cutaneous input is inhibited at the level of the spinal cord when monkeys perform active wrist movement; these authors hypothesized that descending motor commands were responsible for the inhibition of the afferent tactile information. Given that these inhibitions of tactile input occurred in another primate (macaque monkeys), it is certainly possible that similar inhibitions of tactile input occurred in our human participants in the current study when they made active exploratory movements. Comments made by some of our participants during execution of the current task suggest an additional possibility for the poorer shape discrimination that occurred during active exploration. When the surface curvature magnitude in the current experiment was relatively high (e.g., 1.8/m), the shape of the stimulus blocks clearly felt circular (in cross section); when the curvature magnitudes were low, however, the stimuli sometimes felt sinusoidal (a single stimulus block would feel both concave *and* convex, e.g., convex towards the left and concave towards the right). The presence of this shape illusion, which was only reported during active haptic manipulation, would obviously make it difficult for participants to distinguish convex from concave stimuli. All of the coauthors (JRC, JFN, & AK) were able to experience this illusion on at least some sample trials during active haptic exploration. Given this, it was very interesting for us to find that similar subjective experiences have apparently occurred in other studies^32^. The participants of Heller *et al.*^32^ felt circular arcs as raised-line drawings; when asked to draw what they felt, some participants drew sinusoidally-shaped contours (see Fig. 6 of Heller *et al.*). Finally, recent research has demonstrated that the effects of active movement are task dependent^33^,^34^,^35^. Active movements can either facilitate performance, for example on haptic temporal tasks^33^, or they can reduce performance^34^,^35^ for haptic spatial tasks, as was found in the current study.

In addition to evaluating active and passive touch, the current experiment assessed the effects of aging. Overall, there was a substantial and adverse effect of age. The older adults’ curvature discrimination thresholds were 43.8 percent larger than those of the younger adults. Nevertheless, despite this reduction in sensitivity to tactile curvature, the pattern of the older adults’ results was identical to that of the younger adults: the performance exhibited during the passive condition was substantially better (lower thresholds) than the performance obtained for active haptic exploration. The overall superiority for passive curvature discrimination is therefore robust and persists throughout the human lifespan.

## Additional Information

**How to cite this article**: Cheeseman, J. R. *et al.* Dynamic cutaneous information is sufficient for precise curvature discrimination. *Sci. Rep.* **6**, 25473; doi: 10.1038/srep25473 (2016).
